# The predictive value of changes in ^18^F-FDG PET/CT cardiac uptake patterns and metabolic parameters for anthracycline based chemotherapy induced cardiac toxicity in lymphoma patients

**DOI:** 10.1371/journal.pone.0319442

**Published:** 2025-02-27

**Authors:** Runlong Lin, Aijuan Tian, Ying Wang, Xiaomei Wang, Xin Yuan, Jing Yu, Guihua Li, Wenli Xie

**Affiliations:** 1 Department of Nuclear Medicine, The Second Hospital of Dalian Medical University, Dalian, China; 2 Department of Hematology, The Second Hospital of Dalian Medical University, Dalian, China; 3 Department of Health Management Center, The Second Hospital of Dalian Medical University, Dalian, China; 4 Department of Cardiovascular Medicine, The Second Hospital of Dalian Medical University, Dalian, China; IRCCS Ospedale Policlinico San Martino, Genova, Italy, ITALY

## Abstract

**Objective:**

This study aims to examine alterations in positron emission tomography with 2-deoxy-2-[fluorine-18]fluoro-D-glucose integrated with computed tomography (^18^F-FDG PET/CT) heart uptake patterns and metabolic factors before and after anthracycline-based chemotherapy in lymphoma patients, and to investigate the added benefit of oncological ^18^F-FDG PET/CT in chemotherapy-induced heart damage.

**Materials and Methods:**

Between July 2017 and December 2022, lymphoma patients diagnosed at the Second Affiliated Hospital of Dalian Medical University who underwent 6 cycles of anthracycline-based chemotherapy and had baseline and 6-cycle oncological ^18^F-FDG PET/CT scans were included. A total of 366 patients with complete data sets were enrolled. Relevant parameters including blood tests, lipid profile, cardiac biomarkers, lactate dehydrogenase (LDH), erythrocyte sedimentation rate (ESR), albumin (ALB), β2-microglobulin (β2-MG), and cardiac ultrasound findings were collected. Patients were monitored from the initiation of chemotherapy until January 2024, and the occurrence of cancer therapy-related cardiovascular toxicity (CTR-CVT) was documented. Changes in PET/CT heart uptake patterns pre- and post-treatment, along with the presence or absence of CTR-CVT, were used to analyze alterations in left ventricular and epicardial adipose tissue metabolic parameters, as well as changes in echocardiographic parameters. Logistic regression analysis was employed to identify risk factors for CTR-CVT.

**Results:**

Among lymphoma patients who received 6 cycles of anthracycline-based chemotherapy, compared to their initial state, there was a notable decrease in white blood cell count (WBC), neutrophil-to-lymphocyte ratio (NLR), erythrocyte sedimentation rate (ESR), and β2-microglobulin (β2-MG) levels post-treatment. Conversely, albumin (ALB) levels and blood lipid levels significantly rose after treatment. Post-treatment, the maximum standardized uptake value (SUVmax) and mean standardized uptake value (SUVmean) of the left ventricle significantly increased, and the percentage of patients exhibiting no uptake pattern in the left ventricle significantly decreased, while those with diffuse uptake pattern notably increased. Moreover, the count of patients with abnormal cardiac uptake significantly rose post-treatment. Analyzing changes in uptake patterns, the group displaying abnormal changes exhibited an increase in left atrial diameter and a decrease in left ventricular ejection fraction compared to the group with normal changes. The SUVmax of the epicardial adipose tissue was notably higher in the abnormal change group compared to the normal change group. Based on the presence or absence of CTR-CVT, the CTR-CVT group showcased higher left atrial diameter and left ventricular end-systolic diameter, and lower left ventricular ejection fraction compared to the non-CTR-CVT group. Additionally, the SUVmax and SUVmean of the epicardial adipose tissue were higher in the CTR-CVT group than in the non-CTR-CVT group. Left atrial end-systolic diameter, left ventricular ejection fraction, SUVmax of the epicardial adipose tissue, and change in uptake pattern were identified as risk factors for CTR-CVT.

**Conclusion:**

In lymphoma patients treated with anthracycline-based chemotherapy, alterations in ^18^F-FDG PET/CT cardiac uptake patterns and metabolic parameters observed during the follow-up period before and after treatment, as well as changes in epicardial adipose tissue metabolic parameters post-treatment, could serve as predictors for the occurrence of CTR-CVT.

## Introduction

Cancer and heart diseases are the primary causes of mortality, and chemotherapy has substantially enhanced the survival rates of cancer patients by reducing deaths and improving overall prognosis [1]. Nevertheless, the medications utilized in cancer treatment can have adverse effects on patient health, particularly cardiac toxicity, which stands as one of the most detrimental complications of cancer therapy. Hence, close monitoring of cardiac toxicity during treatment is recommended.

Cancer therapy-related cardiovascular toxicity (CTR-CVT) encompasses a spectrum of conditions such as cancer therapy-related cardiac dysfunction (CTRCD), coronary artery disease, valvular heart disease, arrhythmias, hypertension, thrombosis and thromboembolic diseases, peripheral artery disease, bleeding complications, pulmonary hypertension, and pericardial diseases [2]. CTRCD captures the broad spectrum of possible presentations, such as cardiac injury, cardiomyopathy, and heart failure [2]. CTRCD commonly affects 9.3–43.8% of patients undergoing anthracycline-based chemotherapy [3]. Anthracycline-based chemotherapies (AC) are prevalent anti-tumor medications and pivotal components of various chemotherapy protocols, rendering them extensively employed. AC-induced cardiac and endothelial dysfunction exhibit dose-dependent impacts, leading to diverse forms of cardiovascular impairment [4]. Anthracycline drugs induce cardiotoxicity by instigating the binding of reactive oxygen species and topoisomerase IIβ, lipid peroxidation, inflammatory responses, and mitochondrial impairment. Consequently, this prompts apoptosis, necrosis, and interstitial fibrosis of myocardial cells, elevating the risk of coronary endothelial dysfunction, left ventricular dysfunction, and heart failure. These ramifications notably heighten patient mortality rates and impact prognosis [5].

The occurrence of CTR-CVT, particularly CTRCD, hinges on factors such as the cumulative dosage of anthracycline drugs, cardiovascular risk elements, and the duration of follow-up. However, findings from the SUSPOUR study unveiled a negligible alteration in left ventricular ejection fraction (LVEF) by ‒0.03% ± 7.9% over three years, with 85% of patients not meeting CTRCD criteria [6]. Although data concerning early-onset CTRCD during anthracycline-based drug therapy remains scarce, current CTRCD incidence rates are relatively low. Alongside LVEF monitoring, assessing left ventricular global longitudinal strain (LV-GLS) via echocardiography has emerged as a fundamental imaging modality pre-, during-, and post-chemotherapy. Throughout cumulative anthracycline-based drug exposure, LV-GLS deteriorates earlier than LVEF, and a relative LV-GLS reduction exceeding 15% in asymptomatic patients serves as a criterion for asymptomatic CTRCD [7]. However, in numerous investigations, this threshold is gauged by contrasting post-chemotherapy LV-GLS values with baseline strain assessments over an extended period, thus presenting limitations in early CTR-CVT prediction during the anthracycline-based drug cycle [8]. Subcellular scrutiny of myocardial irregularities proves indispensable for the prompt and sensitive identification of cardiac dysfunction induced by anthracycline-based drugs.

To monitor chemotherapy-induced cardiac toxicity, a range of methods are available to assess cardiovascular function, including echocardiography, electrocardiography (ECG), biomarkers, cardiac CT, and cardiac MRI [9]. Nuclear cardiac imaging techniques have demonstrated significant value in tumor diagnosis and treatment evaluation. Specifically, positron emission tomography with 2-deoxy-2-[fluorine-18]fluoro-D-glucose integrated with computed tomography (^18^F-FDG PET/CT) is widely utilized for baseline and follow-up assessments in cancer patients, notably those with lymphoma. The uptake and distribution of ^18^F-FDG in tissues are influenced by glucose levels, fasting duration, and medications. Recent research has indicated that myocardial ^18^F-FDG uptake is not solely reliant on glucose consumption. The retention of the tracer is influenced by the activity of hexose-6-phosphate dehydrogenase (H6PD) in the endoplasmic reticulum, which processes various hexoses, including FDG, thereby initiating the pentose phosphate pathway to maintain NADPH levels in response to oxidative stress induced by chemotherapy [10]. Consequently, ^18^F-FDG PET/CT can serve as an early screening tool for cardiac toxicity in lymphoma patients. Dourado et al. analyzed seventy lymphoma patients who underwent ^18^F-FDG PET/CT examinations and assessed three myocardial uptake parameters—maximum standardized uptake value (SUVmax) of the left ventricle, heart-to-blood pool ratio, and heart-to-liver ratio—at baseline, mid-term, and post-treatment. Their findings revealed a significant increase in myocardial ^18^F-FDG uptake during and after chemotherapy in lymphoma patients, highlighting the sensitivity and reliability of ^18^F-FDG PET/CT as an imaging modality for detecting early metabolic changes indicative of cardiac toxicity [11].

Presently, research on assessing cardiac toxicity related to tumor radio-chemotherapy utilizing ^18^F-FDG PET/CT predominantly focuses on analyzing disparities in myocardial FDG uptake metabolic parameters. However, there exists limited investigation into the precise location, pattern, and significance of myocardial FDG uptake. Thus, this study endeavors to elucidate the supplementary utility of ^18^F-FDG PET/CT in monitoring cardiac toxicity during tumor treatment by scrutinizing alterations in the location, pattern, and metabolic parameters of cardiac uptake before and after chemotherapy.

## Materials and methods

### Study subjects

A total of 366 patients diagnosed with lymphoma and treated with a 6-cycle anthracycline-based chemotherapy regimen at the Second Affiliated Hospital of Dalian Medical University between 1 July 2017 and 31 December 2022 were enrolled in this study. Each patient underwent baseline and post-6-cycle chemotherapy ^18^F-FDG PET/CT scans, and complete case data were available. Data were accessed for research purposes on 30 June 2023. Inclusion criteria were as follows: (1) Pathologically confirmed lymphoma patients undergoing an anthracycline-based chemotherapy regimen, with baseline and post-6-cycle chemotherapy ^18^F-FDG PET/CT imaging; (2) Comprehensive evaluation of cardiac medical history, including echocardiography, ECG, cardiac biomarkers (creatine kinase, troponin), and brain natriuretic peptide (BNP). Exclusion criteria included: (1) Poor quality of PET/CT images; (2) Incomplete collection of patient case data; (3) History of previous tumors and receipt of radiation or chemotherapy; (4) Poorly controlled severe diabetes; (5) Severe liver or kidney dysfunction; (6) Patients with cardiac lesions (e.g., tumors, granulomatous diseases, etc.). This study had approval from the Ethics Committee of the Second Hospital of Dalian Medical University. All enrolled patients provided written informed consent for their participation in the study. The study was conducted according to the Declaration of Helsinki. Additionally, access to information that could potentially identify individual participants post data collection was secured.

### Collection of clinical data

Reviewing outpatient and inpatient medical records, as well as PET/CT examination records, to gather patients’ general clinical data, including age, gender, hypertension, diabetes, cardiac disease history, history of radiation and/or chemotherapy, purpose of PET/CT imaging, laboratory parameters, including White Blood Cell (WBC), Erythrocyte Sedimentation Rate (ESR), Lactate Dehydrogenase (LDH), Albumin (ALB), β2-microglobulin (β2-MG), Total Cholesterol (TC), Triglyceride (TG), High-density lipoprotein cholesterol (HDL-C), Low density lipoprotein cholesterol (LDL-C), calculating Neutrophil/lymphocyte ratio (NLR), final diagnosis outcomes, chemotherapy regimens, etc. Additionally, collect cardiac-related imaging data, such as ECG, echocardiography, coronary artery CT imaging, and coronary angiography results.

### ^18^F-FDG PET/CT examination

We employed the Philips Ingenuity TF PET/CT scanner for the assessments. The ^18^F-FDG was produced and synthesized using the Sumitomo HM-10 cyclotron accelerator and the chemical synthesis module from PET CO., LTD. (Beijing), ensuring a radiochemical purity exceeding 95%. Patients refrained from eating for at least 12 hours before the procedure. Following the administration of ^18^F-FDG at a dosage of 3.7–5.55 MBq/kg, patients rested in a dimly lit room for 60 minutes before undergoing PET/CT scans post-bladder voiding. The scan ranged from the skull base to the foot. Initially, CT scans were performed with parameters set at a voltage of 120 kV, current of 90 mA, rotation speed of 0.75s/rotation, and a matrix of 512 × 512. Subsequently, PET imaging followed with conditions set at a matrix of 144 × 144 and 1-minute acquisition for each bed position, totaling 8–10 bed positions. After attenuation correction and OSEM reconstruction, PET images were co-registered with CT images on the image processing workstation.

### Myocardial glucose uptake analysis

According to the guidelines of the American Society of Nuclear Cardiology (ASNC) and based on previous research, two experienced nuclear medicine doctors reviewed ^18^F-FDG PET/CT images of myocardial glucose uptake retrospectively [12–14]. They classified myocardial glucose uptake patterns into four types based on visual analysis: (1) No uptake, where overall uptake in the left ventricular myocardium was the same as or lower than uptake in the cardiac blood pool; (2) Diffuse uptake, where the left ventricular myocardium showed overall uptake with a fairly even distribution, without focal or significantly higher uptake; (3) Focal uptake, where certain areas of the left ventricular myocardium had higher uptake, while the rest had uptake equal to or lower than the blood pool; (4) Focal uptake on a diffuse uptake background, where certain segments of the myocardium showed higher uptake against a diffuse background of myocardial uptake. Myocardial glucose uptake could be categorized based on its location as left ventricular myocardial uptake, right ventricular myocardial uptake, or atrial myocardial uptake. Based on the patterns, locations, and characteristics of myocardial glucose uptake, it could be classified as either normal or abnormal cardiac uptake. Abnormal cardiac uptake was defined as: (1) Focal or focal uptake on a diffuse uptake in the left ventricle, excluding basal circumferential or semicircular uptake, focal uptake in the papillary muscle, and uniform uptake in the left ventricular lateral wall; (2) Right ventricular uptake higher than left ventricular uptake; (3) Atrial uptake higher than blood pool uptake when there was no or low uptake in the left ventricle; (4) Excluding normal uptake in specific locations, such as lipomatous hypertrophy of the interatrial septum (LHIS) or crista terminalis of the right atrium.

Based on comparison of PET/CT images pre- and post-chemotherapy, changes in cardiac uptake patterns were classified as normal-normal, normal-abnormal, abnormal-normal, or abnormal-abnormal patterns. The normal-normal pattern constituted the normal change group, while the normal-abnormal, abnormal-normal, and abnormal-abnormal patterns comprised the abnormal change group.

### Quantitative analysis of cardiac and epicardial adipose tissue uptake

On the fused PET/CT images, delineation of the epicardial adipose tissue (EAT) region of interest was performed using CT values ranging from ‒190 to ‒45 Hounsfield units (HU) between myocardium and pericardium [15]. We selected the region adjacent to the origin of right coronary artery for measuring the activity of EAT. This site was less affected by ventricular FDG activity [16]. The maximum adipose tissue ROI was outlined on three consecutive cross sections, and the SUVmax of ROI at each layer was recorded respectively, and the maximum value in the three layers was recorded. The MedEx software, provided by Beijing MedEx Technology Co., Ltd., was utilized to measure the SUVmax and the average standardized uptake value (SUVmean) in both the left ventricle and epicardial adipose tissue.

### Clinical follow-up

From the initiation of tumor chemotherapy until January 2024, clinical follow-up was conducted to document CTR-CVT. CTR-CVT was defined as encompassing CTRCD, coronary artery disease, valvular heart disease, arrhythmias, hypertension, thrombosis and thromboembolic diseases, peripheral artery disease, bleeding complications, pulmonary hypertension, and pericardial diseases [2].

### Statistical analysis

We conducted statistical analyses using SPSS Statistics 26.0. The normality of continuous variables was assessed using the Kolmogorov-Smirnov test. Normally distributed data are presented as mean ±  standard deviation (X ± S), while non-normally distributed data are expressed as median (P25, P75). Categorical variables are depicted as frequencies and percentages (%). To compare differences between two independent samples, we utilized the t-test or Mann-Whitney U test, while the chi-square test was employed to compare rates among these samples. Spearman or Pearson correlation analysis was performed to examine the correlation between two independent samples. Multiple logistic regression analysis was utilized to establish a model for variable selection and intergroup predictive factors. The significance threshold was set at P < 0.05.

## Results

### Clinical data of lymphoma patients

This study included a total of 366 patients who met the specified inclusion and exclusion criteria. Among them, there were 185 male patients and 181 female patients. Hodgkin lymphoma was observed in 36 cases, whereas non-Hodgkin lymphoma was identified in 330 cases. Please refer to Table 1 for more details.

**Table 1:** Clinical data of lymphoma patients.

Age (years)	55.58 ± 14.45
Male (number, %)	185(50.55)
BMI (kg/m^2^)	24.30 ± 4.59
Diabetes (number, %)	41(11.20)
Past medical history	
Hypertension (number, %)	77(21.04)
Coronary artery disease (number, %)	14(3.83)
Atrial fibrillation (number, %)	7(1.91)
Heart failure (number, %)	2(0.55)
Pathological classification	
Hodgkin lymphoma (number, %)	36(9.84)
Non-Hodgkin lymphoma (number, %)	330(90.16)
Stage (Ann Arbor-Cotswolds)	
I/II/III/IV	24/77/63/202
Group	
A/B	301/65
Chemotherapy regimen	
(R)-CHOP	281(76.78)
ABVD	35(9.56)
(R-DA)-EPOCH	41(11.20)
DEP	5(1.37)
CVAD	4(1.09)

BMI: Body Mass Index

### Comparison of clinical and cardiac ultrasound parameters before and after treatment in lymphoma patients

Lymphoma patients underwent 6 cycles of chemotherapy, and the clinical and cardiac ultrasound parameters were compared before and after treatment. It was observed that after treatment, the levels of WBC, NLR, ESR, and β2-MG decreased significantly, with statistically significant differences (P < 0.05). After treatment, ALB, TC, TG, HDL-C, LDL-C, LAD, and LVDD significantly increased compared to before treatment, with statistically significant differences (*P* < 0.05). See Table 2.

**Table 2:** Comparison of clinical and cardiac ultrasound parameters before and after treatment in lymphoma patients.

	Before treatment (n = 366)	After treatment (n = 366)	*P* value
Clinical parameters			
WBC (×10^9^/L)	6.13(4.70, 8.06)	4.36(3.27, 5.90)	0.001
NLR	3.28(2.07, 5.42)	2.94(1.82, 4.68)	0.015
ESR (mm/h)	16.00(7.25, 30.00)	12.00(6.00, 20.25)	0.001
LDH (ug/mL)	235.27(183.88, 363.13)	235.11(196.93, 307.61)	0.759
ALB (g/L)	39.49 ± 6.02	41.44 ± 4.61	0.001
β2-MG (mg/L)	3.27 ± 1.98	2.91 ± 0.88	0.005
TC (mmol/L)	4.60 ± 1.26	5.13 ± 0.98	0.001
TG (mmol/L)	1.43 ± 0.87	1.81 ± 0.95	0.001
HDL-C (mmol/L)	1.08 ± 0.38	1.22 ± 0.34	0.001
LDL-C (mmol/L)	2.68 ± 1.06	2.95 ± 0.79	0.001
Cardiac ultrasound			
LAD (mm)	33.11 ± 4.19	33.85 ± 4.24	0.032
LVDD (mm)	43.73 ± 3.99	44.55 ± 3.96	0.013
IVS (mm)	9.34 ± 1.12	9.48 ± 1.13	0.138
LVPW (mm)	9.20 ± 1.02	9.31 ± 1.03	0.218
LVEF (%)	60.30 ± 1.82	59.84 ± 4.01	0.061

WBC: White Blood Cell; NLR: Neutrophil/lymphocyte ratio; ESR: Erythrocyte Sedimentation Rate; LDH: Lactate Dehydrogenase; ALB: Albumin; β2-MG:β2-microglobulin; TC: Total Cholesterol; TG: Triglyceride; HDL-C: High-density lipoprotein cholesterol; LDL-C: Low density lipoprotein cholesterol; LAD: Left atrial diameter; LVDD: left ventricular end diastolic diameter; IVS: interventricular septum; LVPW: Left ventricular posterior wall; LVEF: Left Ventricular Ejection Fraction

### Comparison of cardiac metabolic parameters before and after treatment in lymphoma patients using ^18^F-FDG PET/CT

Lymphoma patients underwent 6 cycles of chemotherapy, and the cardiac metabolic parameters using ^18^F-FDG PET/CT were compared before and after treatment. It was observed that after treatment, both the left ventricular SUVmax and SUVmean significantly increased compared to before treatment, with statistically significant differences (P <  0.05). However, there was no significant difference in EAT SUVmax and EAT SUVmean before and after treatment (P >  0.05). Please refer to Table 3 for details.

**Table 3:** Comparison of cardiac uptake metabolic parameters before and after treatment in lymphoma patients using ^18^F-FDG PET/CT.

	Before treatment (n = 366)	After treatment (n = 366)	*P* value
LV SUVmax	1.90(1.30, 4.90)	3.85(1.80, 7.43)	0.001
LV SUVmean	1.50(1.00, 2.60)	2.20(1.50, 4.10)	0.001
EAT SUVmax	0.93 ± 0.35	0.97 ± 0.34	0.138
EAT SUVmean	0.81 ± 0.30	0.84 ± 0.29	0.224

LV: left ventricular; EAT: epicardial adipose tissue

### Comparison of cardiac uptake in lymphoma patients before and after treatment using ^18^F-FDG PET/CT

Lymphoma patients underwent 6 cycles of chemotherapy, and the cardiac uptake patterns using ^18^F-FDG PET/CT were compared before and after treatment. It was observed that after treatment, there was a significant decrease in the number of patients with no uptake in the left ventricle and a significant increase in the number of patients with diffuse uptake, with statistically significant differences (P <  0.05). Additionally, the number of abnormal cardiac uptakes significantly increased after treatment compared to before treatment, with statistically significant differences (P <  0.05). However, there was no significant change in the proportion of abnormal cardiac uptake sites before and after treatment (P >  0.05). Please refer to Table 4 for details.

**Table 4:** Comparison of cardiac uptake in lymphoma patients before and after treatment using ^18^F-FDG PET/CT.

	Before treatment(n = 366)	After treatment(n = 366)	*P* value
Left ventricular uptake pattern			
No uptake (number, %)	227 (62.02)	150 (40.98)	0.001
Diffuse uptake (number, %)	125 (34.15)	197 (53.83)	0.001
Focal uptake (number, %)	12 (3.28)	17 (4.64)	0.343
Focal uptake on a diffuse uptake background (number, %)	2 (0.55)	2 (0.55)	1.00
Abnormal cardiac uptake (number, %)	17 (4.64)	32 (8.74)	0.027
Abnormal cardiac uptake sites			
Left ventricle (number, %)	9 (2.50)	14 (3.82)	0.289
Right ventricle (number, %)	4 (1.09)	8 (2.18)	0.244
Atrium (number, %)	6 (1.64)	12 (3.28)	0.152

### Changes in cardiac uptake patterns before and after treatment in lymphoma

**Fig 1:**
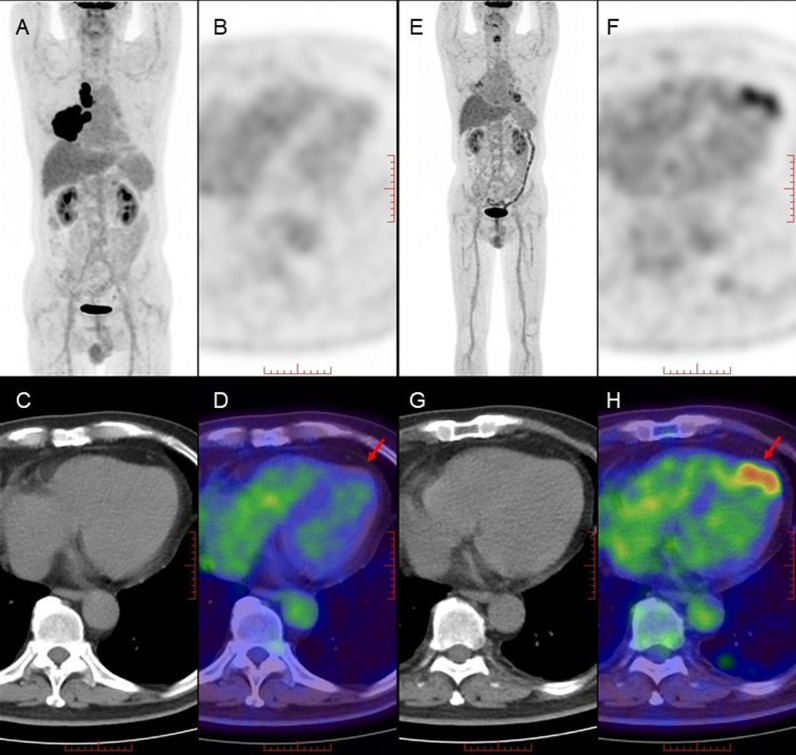
Comparison of changes in cardiac uptake patterns before and after treatment in Lymphoma.

The alterations in cardiac uptake patterns before and after treatment in lymphoma were assessed using ^18^F-FDG PET/CT. The variations in uptake patterns in the left ventricle, right ventricle, and atrium are presented in Table 5. Fig 1 illustrates an instance of a lymphoma patient displaying abnormal uptake in the left ventricle (highlighted by the red arrow) after completing 6 cycles of chemotherapy.

**Table 5:** Changes in cardiac uptake patterns before and after treatment in lymphoma using ^18^F-FDG PET/CT.

Uptake Pattern Changes	Number (%)
Left Ventricle	
normal-normal	348 (95.08)
normal-abnormal	9 (2.46)
abnormal-abnormal	5 (1.37)
abnormal-normal	4 (1.09)
Right Ventricle	
normal-normal	358 (97.81)
normal-abnormal	4 (1.09)
abnormal-abnormal	4 (1.09)
Atrium	
normal-normal	353 (96.45)
normal-abnormal	7 (1.91)
abnormal-abnormal	5 (1.37)
abnormal-normal	1 (0.27)

Male, 67 years old, diagnosed with diffuse large B-cell lymphoma, treated with R-CHOP for 6 cycles. Figs 1A–1D (MIP image, axial PET, CT and PET/CT fusion image): No abnormal uptake in the heart before lymphoma treatment (red arrow). Figs 1E–1H (MIP image, axial PET, CT and PET/CT fusion image): Focal abnormal uptake in the left ventricular apex after lymphoma treatment (red arrow).

### Analysis of different cardiac uptake pattern changes after treatment using ^18^F-FDG PET/CT in lymphoma patients

Following lymphoma treatment, patients were categorized into two groups based on changes in cardiac uptake patterns using ^18^F-FDG PET/CT. The normal change group comprised 330 patients, while the abnormal change group consisted of 36 patients. Cardiac ultrasound results were then compared between these groups. The analysis revealed that the abnormal change group exhibited a higher left atrial diameter and lower left ventricular ejection fraction compared to the normal change group, with statistically significant differences (P <  0.05), as demonstrated in Table 6.

**Table 6:** Comparison of cardiac ultrasound in lymphoma patients with different ^18^F-FDG PET/CT cardiac uptake pattern changes after treatment.

	normal change group (n = 330)	abnormal change group (n = 36)	*P* value
LAD (mm)	33.67 ± 4.27	35.39 ± 3.70	0.039
LVDD (mm)	44.47 ± 3.94	45.23 ± 4.14	0.326
IVS (mm)	9.45 ± 1.13	9.67 ± 1.16	0.341
LVPW (mm)	9.30 ± 1.02	9.33 ± 1.05	0.874
LVEF (%)	60.04 ± 1.65	58.17 ± 11.36	0.018

LAD: Left atrial diameter; LVDD: left ventricular end diastolic diameter; IVS: interventricular septum; LVPW: Left ventricular posterior wall; LVEF: Left Ventricular Ejection Fraction

The comparison between the normal change group and abnormal change group indicated that the SUVmax of the epicardial adipose tissue was notably higher in the abnormal change group compared to the normal change group, with statistically significant differences (P <  0.05), as outlined in Table 7.

**Table 7:** Comparison of metabolic parameters in lymphoma patients with different ^18^F-FDG PET/CT cardiac uptake pattern changes after treatment.

	normal change group (n = 330)	abnormal change group (n = 36)	*P* value
LV SUVmax	4.00 (1.80, 7.50)	2.80 (1.73, 7.20)	0.540
LV SUVmean	2.20 (1.50, 4.15)	2.05 (1.50, 3.88)	0.494
EAT SUVmax	0.96 ± 0.34	1.11 ± 0.34	0.010
EAT SUVmean	0.83 ± 0.29	0.93 ± 0.31	0.059

LV: left ventricular; EAT: epicardial adipose tissue

### Analysis of different CTR-CVT groups after lymphoma treatment

**Fig 2:**
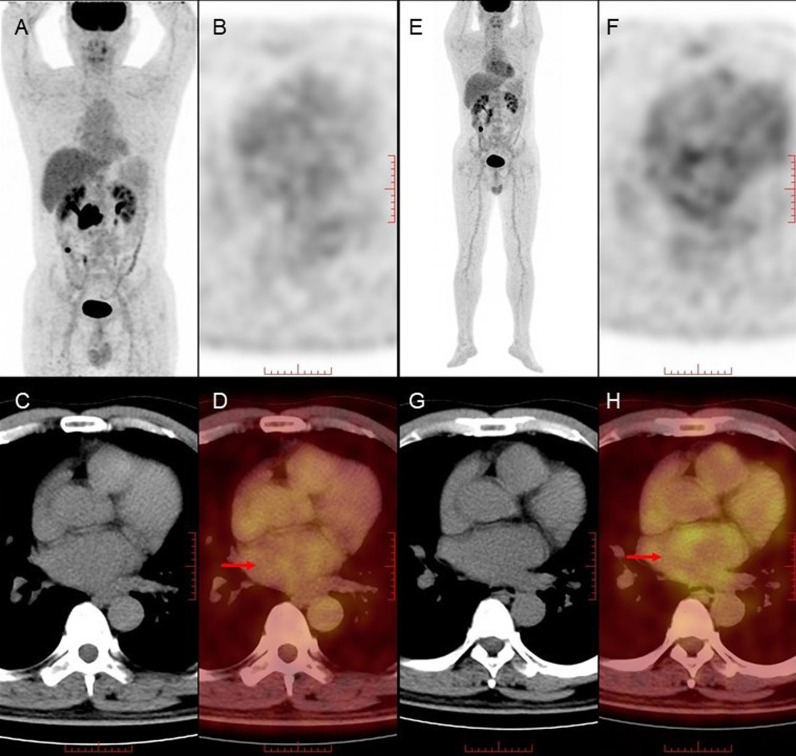
Abnormal Cardiac Uptake in the Atrium of a Newly Developed Atrial Fibrillation Patient After Lymphoma Treatment.

After lymphoma treatment, patients were categorized based on the presence or absence of CTR-CVT. The CTR-CVT group comprised 41 patients, while the non-CTR-CVT group consisted of 325 patients. Subsequently, cardiac ultrasound results were compared between these groups. The analysis revealed that the CTR-CVT group displayed higher left atrial diameter and left ventricular end-diastolic diameter compared to the non-CTR-CVT group. Additionally, the left ventricular ejection fraction was lower in the CTR-CVT group compared to the non-CTR-CVT group, with statistically significant differences (P <  0.05), as depicted in Table 8. Fig 2 illustrates an example of a newly developed atrial fibrillation patient after lymphoma treatment, showcasing changes in cardiac uptake patterns before and after treatment.

**Table 8:** Comparison of cardiac ultrasound in lymphoma patients with different CTR-CVT groups after treatment.

	CTR-CVT		*P* value
**Yes (n = 41)**	**No (n = 325)**	
LAD (mm)	35.19 ± 4.27	33.62 ± 4.20	0.028
LVDD (mm)	46.74 ± 4.18	44.16 ± 3.80	0.001
IVS (mm)	9.73 ± 1.12	9.43 ± 1.02	0.117
LVPW (mm)	9.51 ± 1.04	9.27 ± 1.02	0.168
LVEF (%)	57.17 ± 9.54	60.31 ± 1.28	0.001

LAD: Left atrial diameter; LVDD: left ventricular end diastolic diameter; IVS: interventricular septum; LVPW: Left ventricular posterior wall; LVEF: Left Ventricular Ejection Fraction

Male, 51 years old, diagnosed with diffuse large B-cell lymphoma, treated with R-CHOP for 6 cycles. Figs 2A–D (MIP image, axial PET, CT and PET/CT fusion image): No abnormal uptake in the atrium before lymphoma treatment (red arrow). Figs 2E–H (MIP image, axial PET, CT and PET/CT fusion image): Abnormal uptake in the left atrium higher than the blood pool uptake after lymphoma treatment (red arrow).

Following the categorization of patients based on the presence or absence of CTR-CVT, it was discovered that the proportion of abnormal uptake pattern changes was notably higher in the CTR-CVT group compared to the non-CTR-CVT group. Furthermore, the SUVmax and SUVmean of the epicardial adipose tissue were significantly elevated in the CTR-CVT group compared to the non-CTR-CVT group, with statistically significant differences (P <  0.05), as outlined in Table 9.

**Table 9:** Comparison of metabolic parameters in lymphoma patients with different CTR-CVT groups after treatment.

	CTR-CVT		
	**Yes (n = 41)**	**No (n = 325)**	*P* value
Abnormal uptake pattern changes, n (%)	10 (24.39)	26 (8.00)	0.001
LV SUVmax	4.40 (1.60, 8.60)	3.70 (1.80, 7.40)	0.555
LV SUVmean	2.30 (1.30, 4.60)	2.20 (1.50, 4.05)	0.779
EAT SUVmax	1.12 ± 0.39	0.95 ± 0.33	0.003
EAT SUVmean	0.93 ± 0.32	0.83 ± 0.29	0.038

LV: left ventricular; EAT: epicardial adipose tissue

### Logistic regression analysis of risk factors for CTR-CVT after lymphoma treatment

Logistic regression analysis was performed to identify the risk factors for CTR-CVT after lymphoma treatment. The results revealed that left ventricular diameter [OR = 1.177 (95% CI: 1.038, 1.335)], left ventricular ejection fraction [OR = 0.537 (95% CI: 0.382, 0.756)], epicardial adipose tissue (EAT) SUVmax [OR = 2.668 (95% CI: 0.842, 8.457)], and abnormal uptake pattern changes [OR = 3.564 (95% CI: 1.156, 10.994)] were identified as risk factors for CTR-CVT in lymphoma patients after treatment. Refer to Table 10 for further details.

**Table 10:** Logistic regression analysis of different risk factors for CTR-CVT after lymphoma treatment.

	B	S.E.	P	Exp (B)
LVD	0.163	0.064	0.011	1.177 (1.038, 1.335)
LVEF	‒0.621	0.174	0.001	0.537 (0.382, 0.756)
EAT SUVmax	0.981	0.589	0.096	2.668 (0.842, 8.457)
Uptake pattern changes	1.271	0.575	0.027	3.564 (1.156, 10.994)

## Discussion

As cancer survival rates continue to improve, the cardiovascular side effects of cancer treatments significantly impact patient prognosis. Previous studies have highlighted a clear correlation between factors such as pre-existing cardiovascular diseases, cardiovascular risk factors, genetic predisposition, treatment regimens, age, and the risk of cardiovascular complications post-cancer treatment [17]. The spectrum of CTR-CVT varies widely, ranging from asymptomatic reversible changes to life-threatening complications, encompassing heart failure, acute coronary syndrome, arrhythmias, valvular heart disease, pericardial disease, myocarditis, and thromboembolic events [18]. Consequently, there’s a pressing need for in-depth exploration of the mechanisms underlying cancer and cancer therapy-induced cardiovascular diseases, establishment of appropriate diagnostic protocols, early identification of CTR-CVT, and implementation of effective treatment and prevention strategies. These endeavors constitute the primary focus of current research in onco-cardiology and serve as the cornerstone for interdisciplinary discussions among cardiology experts and oncology treatment teams.

Cancer patients, particularly those at high risk for cardiovascular complications, necessitate standardized monitoring of CTR-CVT throughout the treatment journey to promptly identify associated side effects. This entails conducting baseline risk assessments prior to treatment initiation, monitoring for acute complications during treatment, and ensuring long-term follow-up for late-stage cardiovascular effects [19]. Pre-treatment assessment involves gathering medical history, conducting physical examinations, performing ECGs, assessing cardiac biomarkers, including N-terminal pro-brain natriuretic peptide (NT-proBNP), and conducting echocardiography. Moreover, comprehensive documentation of the cancer treatment plan, encompassing conventional chemotherapy, targeted therapy, immunotherapy, or radiation therapy, is imperative. During treatment, cardiac oncology monitoring is tailored according to the patient’s cardiac risk profile, with echocardiography serving as the preferred imaging modality for tracking cardiac damage in cancer patients. In cases where cardiac toxicity is suspected, additional diagnostic modalities such as cardiac magnetic resonance imaging or cardiac catheterization may be warranted. Particularly, patients at elevated risk for late-stage complications from radiation therapy may undergo coronary angiography to evaluate coronary artery disease [19].

Nuclear imaging techniques can unveil metabolic alterations at the molecular level and play a pivotal role in detecting myocardial cell toxicity before irreversible damage occurs. ^18^F-FDG PET/CT can identify abnormal myocardial cell survival and assess myocardial inflammatory reactions induced by cancer treatment, though often necessitating suppression of myocardial glucose metabolism [20]. Anthracycline drugs can disrupt normal mitochondrial oxidative metabolism, thereby causing aberrant energy metabolism in myocardial cells. Anthracyclines’ cardiotoxicity involves an accelerated generation of reactive oxygen species. This oxidative damage has been found to accelerate the expression of hexose-6P-dehydrogenase (H6PD), that channels glucose-6-phosphate (G6P) through the pentose phosphate pathway (PPP) confined within the endoplasmic/sarcoplasmic reticulum (SR) [21]. The direct correlation between cardiac FDG uptake and oxidative stress indexes supports the potential role of FDG-PET as an early biomarker of Doxorubicin oxidative damage [22]. Consequently, ^18^F-FDG PET/CT is well-suited for detecting anthracycline-induced myocardial cell damage [23].

From an economic and practical standpoint, only a small subset of cancer patients undergoes dedicated ^18^F-FDG cardiac metabolic or inflammation imaging. However, can oncologic ^18^F-FDG imaging serve as a means to evaluate the toxic effects of tumor drugs on the cardiovascular system while concurrently assessing tumor diagnosis and efficacy? Dourado et al. conducted a study comparing the cardiac uptake SUV values of 70 lymphoma patients before and after treatment using ^18^F-FDG PET/CT imaging and observed a significant increase in cardiac uptake of ^18^F-FDG post-treatment [11]. Sarocchi et al. found DOX-containing chemotherapy causes an increase in cardiac ^18^FDG uptake, which is associated with a decline in LVEF [24]. Similarly, our research demonstrated a notable increase in left ventricular ^18^F-FDG uptake in lymphoma patients treated with anthracycline-based chemotherapy compared to pre-treatment levels. In our study, abnormal cardiac uptake was classified based on location, revealing changes in uptake in the left ventricle, right ventricle, and atrium. Kim et al. investigated 121 breast cancer patients and compared right ventricular wall ^18^F-FDG uptake before and after treatment using ^18^F-FDG PET/CT. They discovered that breast cancer patients receiving anthracycline drugs or trastuzumab exhibited increased uptake in the right ventricular wall, which correlated with cardiac toxicity [25]. Hence, abnormal uptake and alterations in SUV values in different cardiac regions may reflect the cardiotoxic effects of tumor drugs to some extent. Additionally, Vinogradskiy et al. explored the relationship between radiation dose to the heart and overall survival in lung cancer patients undergoing chemotherapy. They found that changes in SUV in the heart post-treatment were significant factors for dose response and predictive of overall survival. This suggests that alterations in cardiac metabolism may serve as early predictive indicators of clinical outcomes [26].

In our study, we observed a significant decrease in the proportion of patients exhibiting no uptake pattern in the left ventricle after treatment, while those with a diffuse uptake pattern significantly increased. This suggests that post-chemotherapy, there’s a heightened myocardial uptake of ^18^F-FDG among patients, albeit possibly representing physiological uptake in the case of diffuse uptake. Subsequently, we noted a notable increase in the number of patients displaying abnormal uptake patterns post-treatment, indicating potential abnormal myocardial ^18^F-FDG metabolism during the treatment process. These uptake patterns and changes in SUV may indeed mirror the cardiotoxic effects of tumor drugs. Based on the alterations in patterns before and after treatment, we segregated patients into the normal change group and the abnormal change group. Remarkably, we observed that patients in the abnormal change group exhibited an augmented left atrial diameter and reduced left ventricular ejection fraction post-treatment compared to the normal change group, suggesting potential impairment of cardiac function in the abnormal change group. Furthermore, in the abnormal change group, the EAT SUVmax surpassed that of the normal change group, indicating shifts in the uptake of ^18^F-FDG in the epicardial adipose tissue. Epicardial adipose tissue has been implicated in the release of inflammatory factors, fostering atrial remodeling. Activation of inflammatory cells triggers the release of numerous cytokines, upregulates glucose transporters, and enhances the uptake of ^18^F-FDG by inflammatory cells [27]. These findings may signify an association with damage and cardiac inflammatory activity.

Based on the occurrence of CTR-CVT during the treatment process, our findings revealed that patients in the CTR-CVT group exhibited larger left atrial diameter and left ventricular end-systolic diameter compared to those in the non-CTR-CVT group, along with a lower left ventricular ejection fraction. Additionally, the EAT SUVmax and EAT SUVmean post-treatment were higher in the CTR-CVT group than in the non-CTR-CVT group, indicating that the cardiotoxicity of tumor drugs had repercussions on the left atrium, left ventricle, and ejection fraction. Through regression analysis, we identified several risk factors for CTR-CVT, including left ventricular end-systolic diameter, ejection fraction, EAT SUVmax, and changes in uptake patterns post-treatment. Although left ventricular end-systolic diameter and ejection fraction hold predictive potential for future cardiac toxicity, their changes during treatment may be subtle and fall within the normal range, making them easily overlooked. However, during the follow-up of tumor lesions using ^18^F-FDG PET/CT, alterations in cardiac uptake patterns and epicardial adipose tissue uptake values, particularly noteworthy differences observed in visual analysis of cardiac uptake patterns, may signify the likelihood of future cardiovascular toxicity. This highlights the additional value of predicting cardiovascular toxicity in oncologic imaging.

This study is retrospective in nature, and the fasting protocol for PET/CT scans is tailored towards oncology, differing from the fasting protocol used for evaluating cardiac inflammation imaging. Although a prolonged fasting period (>12 hours) is mandated for tumor imaging patients at our center, it may not entirely suppress the physiological uptake of myocardium for ^18^F-FDG PET/CT. Consequently, further validation of lesions exhibiting changes in cardiac uptake patterns is warranted through prospective experiments or animal studies to elucidate pathological or histological characteristics. However, notwithstanding these limitations, the current research findings instill optimism.

## Conclusion

In our study, we suggest that during the follow-up process of oncologic ^18^F-FDG PET/CT, early prediction of future CTR-CVT may be feasible by observing alterations in cardiac uptake patterns and metabolic parameters of the heart and epicardial adipose tissue. This is a new aid for early identification and intervention of cardiac-related complications in oncology patients to improve the quality of survival.
